# Detection and quantification of *Mycobacterium tuberculosis* antigen CFP10 in serum and urine for the rapid diagnosis of active tuberculosis disease

**DOI:** 10.1038/s41598-021-98471-1

**Published:** 2021-09-28

**Authors:** Marva Seifert, Eva Vargas, Victor Ruiz-Valdepeñas Montiel, Joseph Wang, Timothy C. Rodwell, Antonino Catanzaro

**Affiliations:** 1 Division of Pulmonary, Critical Care and Sleep Medicine, Department of Medicine, University of California San Diego, 9500 Gilman Drive, La Jolla, CA 92093 USA; 2 Department of Nanoengineering, University California San Diego, 9500 Gilman Drive, La Jolla, CA 92093 USA

**Keywords:** Tuberculosis, Diagnostic markers, Molecular medicine

## Abstract

Outside of the ongoing COVID-19 pandemic, tuberculosis is the leading cause of infectious disease mortality globally. Currently, there is no commercially available point-of-care diagnostic that is rapid, inexpensive, and highly sensitive for the diagnosis of active tuberculosis disease. Here we describe the development and optimization of a novel, highly sensitive prototype bioelectronic tuberculosis antigen (BETA) assay to detect tuberculosis-specific antigen, CFP10, in small-volume serum and urine samples. In this proof-of-concept study we evaluated the performance of the BETA assay using clinical specimens collected from presumptive tuberculosis patients from three independent cohorts. Circulating CFP10 antigen was detected in ALL serum (n = 19) and urine (n = 3) samples from bacteriologically confirmed tuberculosis patients who were untreated or had less than one week of treatment at time of serum collection, successfully identifying all culture positive tuberculosis patients. No CFP10 antigen was detected in serum (n = 7) or urine (n = 6) samples from individuals who were determined to be negative for tuberculosis disease. Additionally, antigen quantification using the BETA assay of paired serum samples collected from tuberculosis patients (n = 8) both before and after treatment initiation, indicate consistently declining within-person levels of CFP10 antigen during treatment. This novel, low-cost assay demonstrates potential as a rapid, non-sputum-based, point-of-care tool for the diagnosis of tuberculosis disease.

## Introduction

While SARS-CoV-2 has temporarily displaced *Mycobacteria tuberculosis* (*Mtb*) as the leading cause of infectious disease mortality worldwide, it is expected that the COVID-19 pandemic might ultimately add 400,000 excess tuberculosis (TB) deaths to the annual TB mortality of 1.4 million TB deaths reported in 2019^1^. The World Health Organization (WHO) estimates that approximately 10 million individuals develop active TB annually, but 30% of these (3 million) are never diagnosed or reported^2^. This gap in diagnosis is due in part to the lack of a rapid, highly sensitive, and inexpensive point-of-care TB diagnostic solution that can be deployed at the lowest levels of the healthcare system. Treatment delays due to these gaps in diagnosis are linked to poor treatment outcomes and contribute to continued transmission.

The most frequently used TB diagnostic for active disease detection in endemic regions remains sputum smear microscopy, a method introduced over a century ago. The method is inexpensive and rapid, but has limited sensitivity among immunocompetent individuals (40–60%), even lower sensitivity (20–40%) among individuals living with HIV, and low specificity in regions with high non-tuberculous mycobacteria (NTM) prevalence^3,4^. Other commercially available diagnostic solutions used to detect *Mtb* in sputum, include: (1) culture-based methods which are sensitive and specific, but can take weeks to produce results, are expensive, and require advanced laboratory capacity; and (2) molecular-based, nucleic acid amplification tests (NAAT), which are rapid, highly sensitive and specific, but also complex and costly, and at 10–30 USD/test in the public sector globally and 30–175 USD/test in the private sector in high burden countries can be cost prohibitive^5,6^. Furthermore, most commercial diagnostic solutions are optimized for sputum, resulting in reduced sensitivity in patients who have difficulty producing sputum, such as children, as well as people living with HIV, who are also more likely to have paucibacillary disease^7,8^. Detection of extrapulmonary TB disease is also severely limited by sputum optimized diagnostics as the sensitivity of these diagnostics can be highly variable in non-sputum sample types^9,10^.

A promising alternative to identifying *Mtb* in sputum is the detection of circulating *Mtb*-specific antigens in easily sampled body fluids such as blood and urine. While many novel *Mtb*-specific antigen targets continue to be identified and evaluated, the most frequently investigated antigens include Lipoarabinomannan (LAM), 6-kDa early secreted antigenic target (ESAT6) and 10 kDa culture filtrate protein (CFP10), the latter two which are secreted during bacterial replication and are commonly identified in culture supernatant^11–14^. Commercially available urine-based antigen detection assays have exclusively focused on LAM as a detectable antigen and have demonstrated moderate sensitivity among immunocompromised individuals, however, these LAM assays continue to demonstrate poor sensitivity (< 70%) in general TB populations^5,6,11^, and a 2020 study comparing two urine-based LAM assays (“AlereLAM” and “FujiLAM”) for diagnosis of TB in children showed 31% and 65% sensitivity respectively^15^. Research-based assays for detecting *Mtb* antigens ESAT6 and CFP10 in both urine and serum have been used to successfully identify patients with active TB; however, their technological complexity has impeded efforts to translate these antigen detection approaches into point-of-care diagnostic solutions^16–19^.

Recent technological advances in electrochemical biosensors, including novel detection methods and advanced fabrication, have resulted in the development of ultra-sensitive bioelectronic immunoassays. These potential point-of-care solutions offer distinct advantages over other approaches, such as miniaturization and low cost consumables, portability, small-volume sample requirements, and non-expert workflows^20^. Their application for detection of *Mtb*-specific antigens, specifically CFP10, is still largely unexplored; however, two bioelectronic immunoassays have been previously described: one based on the use of quantum dots with gold-tagged antibodies, and another involving graphene nanocomposite surface with nanoparticle labeled antibodies^21,22^. While the limit of detection (LOD) of CFP10 was in the low nano-molar (nM) range for both assays, the complexity of these prototype assays and their sensor preparation are significant obstacles to their future potential as low cost, scalable point-of-care TB diagnostics.

**Figure 1:**
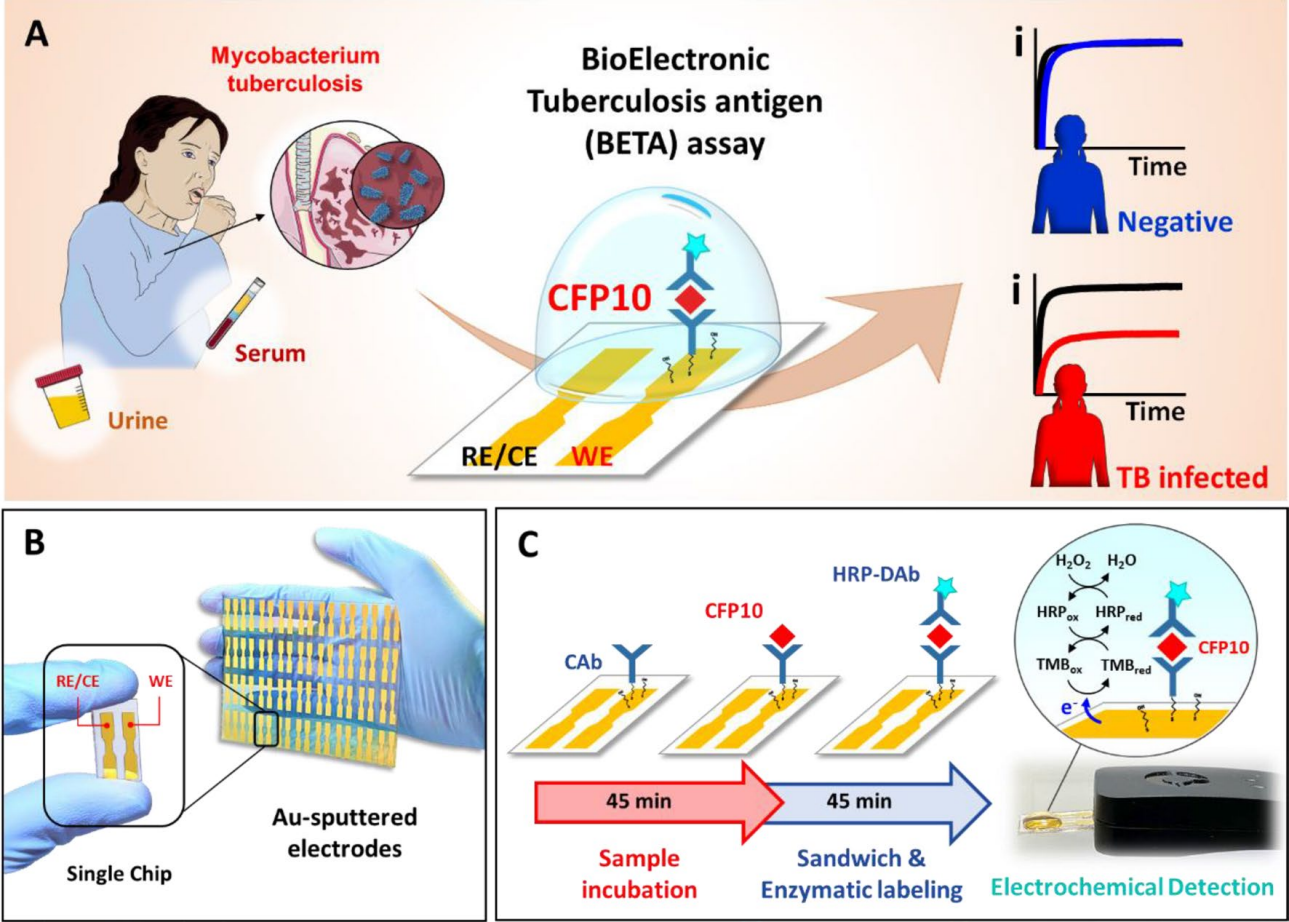
Schematic of a novel bioelectronic TB antigen (BETA) assay showing the workflow and function. (**A**) Image of the sensor with two gold-sputtered electrodes, one employed as working electrode (WE) and one as reference/counter (RE/CE) electrode. (**B**) Schematic of the immunoreaction and transduction processes: a 2-step sandwich-type immunoassay, based on the sequential 45 min incubations of unprocessed clinical sample and the HRP-tagged detector antibody (HRP-DAb), and the electrochemical reaction of the H_2_O_2_/HRP/TMB redox probe that generates current. Illustrations are provided by Servier Medical Art (https://smart.servier.com/) licensed under a Creative Commons Attribution 3.0 Unported License.

In this proof-of-concept study, we demonstrate the successful development, optimization, and potential clinical application of a novel, rapid bioelectronic tuberculosis antigen (BETA) immunoassay for reproducible, highly sensitive detection and quantification of CFP10 in unprocessed small-volume clinical serum and urine samples (Fig. 1). To our knowledge, this is the first time a bioelectronic immunoassay has been successfully used to detect CFP-10 directly from unprocessed clinical urine and serum samples. This quantitative BETA assay, with its low cost-of-goods, simple workflow, small-volume clinical sample requirement, and rapid time to result, has the potential to be deployed as a point-of-care diagnostic solution and treatment response monitoring tool at the lowest levels of the healthcare system.

## Results

### Sensor and assay protocol optimization

Sensor and assay parameters were assessed by comparing the ratio of amperometric results from positive control (“S”—signal) and negative control (“B”—blank) runs, optimizing for highest S/B ratio. Supplementary Figs. S1 and S2 show the S/B ratios for each condition and final resulting Nyquist curves, respectively. Assay parameters were then re-optimized for serum matrix (Fig. S3) with Table S1 showing final parameters selected for buffer and serum assays. Background amperometric responses of the serum-optimized assay for negative control buffer, serum and urine indicated that negative control serum had a nearly four-fold lower electrochemical background noise compared to buffer (Fig. S4a), likely due to partial electrode surface biofouling, typically observed in complex fluids^23,24^. In contrast, negative control urine matrix produced higher electrochemical background signals, likely due to the higher non-specific adsorption of biomolecules present in urine (Fig. S4a). To reduce non-specific adsorption for the urine matrix, sensor electrodes were incubated for 30 min in Gibco® horse serum (ThermoFisher Scientific, MA) as a pre-conditioning step before running urine samples. This significantly improved the S/B ratio for urine (see Fig. S4b)^25^.

### Assay calibration and limit of detection

**Figure 2:**
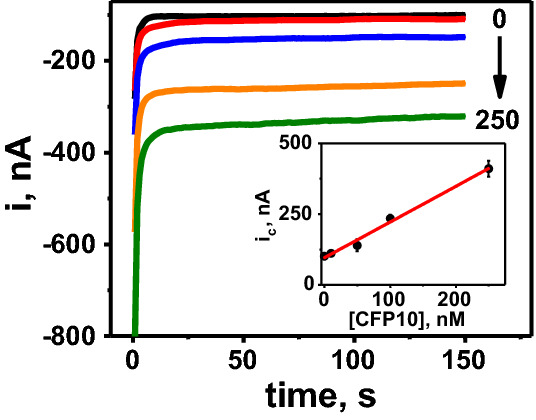
Electrochemical performance and characterization of the buffer optimized bioelectronic TB antigen (BETA) assay using CFP10 spiked into negative control buffer as sample. Chronoamperograms and calibration plot obtained from the analysis of 0.0, 10, 50, 100 and 250 nM CFP10 standards spiked into buffer. Current intensity (i) and cathodic current intensity (i_c_) measured in nA.

**Figure 3:**
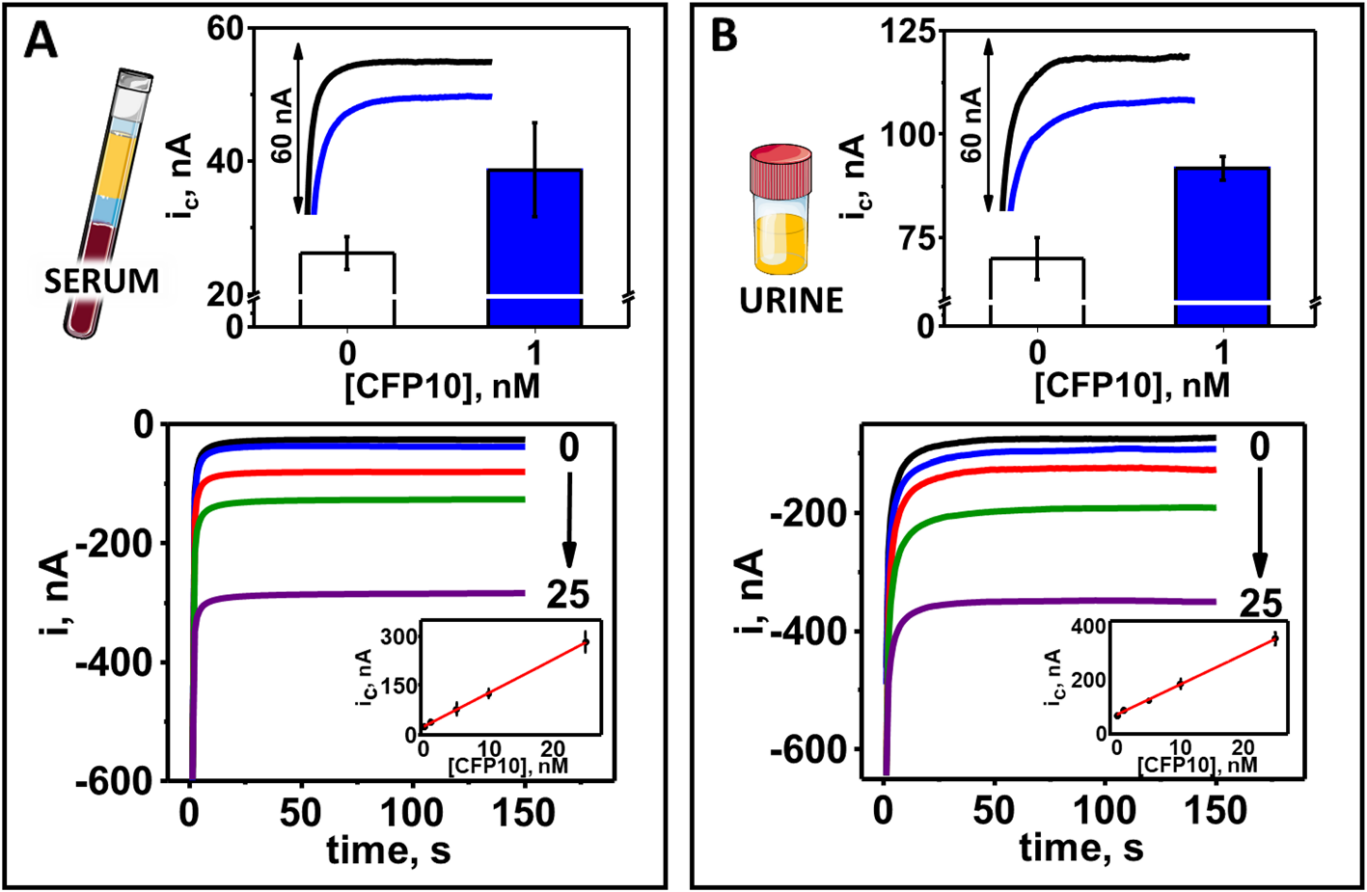
Electrochemical performance and characterization of the serum and urine optimized bioelectronic TB antigen (BETA) assay using CFP10 spiked into negative control serum and urine as samples. Chronoamperometric responses and calibration plots obtained from the analysis of 0.0 (black lines and white bars), 1.0 (blue lines and bars), 5.0 (red lines), 10 (green lines) and 25 nM (purple lines) CFP10 standard spiked into human serum (**A**) and urine (**B**). Current intensity (i) and cathodic current intensity (i_c_) measured in nA. Illustrations are provided by Servier Medical Art (https://smart.servier.com/) licensed under a Creative Commons Attribution 3.0 Unported License.

We produced calibration curves for the optimized BETA assay in buffer (Fig. 2), serum and urine (Fig. 3) to establish matrix-specific baseline analytical performance. See Table 1 for resulting calibration curve parameters. Based on these calibration curves, the estimated LOD for detection of *Mtb* CFP10 antigen in serum was 0.4 nM (44 pg in a 10 µL clinical sample droplet) and 0.7 nM in urine (Table 1).

**Table 1:** Analytical calibration curve characteristics of the optimized bioelectronic TB antigen (BETA) assay for the detection of different concentrations of CFP10 standards prepared in buffer, charcoal stripped (4×) human serum and pooled human urine.

Variable	Buffer	Serum	Urine
R^2^	0.9914	0.9997	0.9985
Slope (nA nM^−1^)	1.3 ± 0.2	10.2 ± 0.2	11.4 ± 0.3
Intercept (nA)	95 ± 26	27 ± 2	74 ± 3
LOD (nM)	3	0.4	0.7
LOD (pg)^a^	330	44	77

^a^Amount of CFP10 antigen detected in a 10 µL simple droplet used for all assay runs.

### Sensor reproducibility and stability

**Figure 4:**
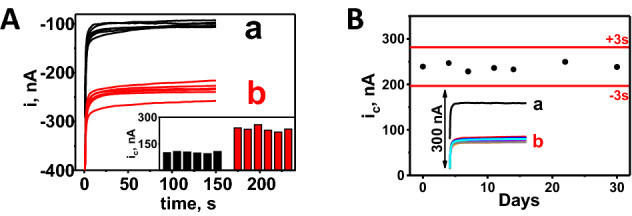
Reproducibility and stability of a bioelectronic TB antigen (BETA) assay in buffer. (**A**) Reproducibility of the BETA assay using both phosphate buffer negative controls ×6 (a, black lines and black bars) and phosphate buffer spiked with 100 nM *Mtb* CFP10 antigen ×6 positive controls (b, red lines and bars). (**B**) Amperometric results from 3 × replicate BETA assay runs on each of 7 days over a 30-day period after fabrication and storage at 4 °C, using buffer spiked with 100 nM CFP10 as a positive control. Current intensity (i) and cathodic current intensity (i_c_) measured in nA.

The relative standard deviation (RSD) of amperometric results from a batch of 12 optimized sensors run with positive control (6 × 100 nM of CFP10 in PB) and negative control samples (6 × PB), was 5.9% and 5.2% respectively (Fig. 4A) indicating good reproducibility of the assay. Sensor stability was assessed periodically over a 30 day period. Amperometric responses ranged from 228.4 to 246.9 nA, and no systematic decrease was observed, indicating acceptable stability during observation period (see Fig. 4B).

### Analytical performance using clinical samples

In total we evaluated 33 serum and 10 urine samples from three independent cohorts of patients considered at risk for active pulmonary TB or drug resistant disease (see Table 2). Average age of participants included in the study was 42 (range 24–82), 21 (84%) were male, and two participants diagnosed with TB were also living with HIV. BETA assay results from patients diagnosed with pulmonary TB indicated that circulating CFP10 antigens were detected in ALL serum (n = 19) and urine (n = 3) samples from positive TB patients who were untreated at time of serum collection or had < 1 week of treatment. Conversely, serum (n = 7) and urine (n = 6) samples collected from patients at risk for TB, but who were TB negative by culture, AFB and/or GeneXpert had CFP10 signals that were not significantly different from negative controls. TB patients with a record of ≥ 1 week treatment produced results both above and below the negative control threshold, with values mostly inversely correlated with days of treatment. The resulting preliminary sensitivity and specificity of TB detection among participants with < 1 week of treatment compared to TB negative was therefore 100% (95% CI 81–100%) and 100% (95% CI 72–100%), respectively.

**Table 2:** Amperometric response and estimated concentration of *Mtb* CFP10 antigen in clinical serum and urine samples collected from microbiologically confirmed TB positive and TB negative patients using the BETA assay.

	Patient ID#	Days on treatment	Amperometric response (-I, nA)	CFP10 concentration (nM)
**Serum samples**				
TB negative	A1	n/a	32.8 ± 5.5	ND^a^
	A2	n/a	42.2 ± 2.3	ND
	A3	n/a	43.8 ± 3.3	ND
	A4	n/a	43.8 ± 2.4	ND
	A5	n/a	43 ± 1.2	ND
	A6	n/a	40.4 ± 5.5	ND
	B1	n/a	44.1 ± 0.3	ND
TB positive	A7	0	103.9 ± 1.5	5.9 ± 0.4
	A8	0	74.9 ± 4.5	3.8 ± 0.9
	A9	1	65.4 ± 0.1	1.8 ± 0.3
	A10	14	47.3 ± 4.9	ND
	B2	0	102.9 ± 2.6	5.8 ± 0.6
	B3	0	88.3 ± 1.3	4.5 ± 0.3
	B4	2	88.9 ± 1	4.5 ± 0.8
	B5	3	83.1 ± 1.3	4.0 ± 0.3
	B6	4	78.8 ± 0.2	3.6 ± 0.2
	B7	6	84.4 ± 1.8	4.1 ± 0.4
	C1	0	82 ± 2.3	3.9 ± 0.5
	C1	5	66.6 ± 1.2	2.4 ± 0.9
	C2	0	63.9 ± 2.2	2.2 ± 0.5
	C2	6	56 ± 2.1	1.5 ± 0.5
	C3	0	52.5 ± 2.2	1.1 ± 0.5
	C3	7	33.9 ± 3.1	ND
	C4	0	101.2 ± 4.8	5.7 ± 0.7
	C4	12	72.8 ± 3.2	3.0 ± 0.9
	C5	0	58.2 ± 1.3	1.7 ± 0.3
	C5	13	44.2 ± 2	ND
	C6	0	67.3 ± 2	2.5 ± 0.5
	C6	14	42.8 ± 3.4	ND
	C7	0	62.6 ± 3.1	2.1 ± 0.7
	C7	14	40.8 ± 0.4	ND
	C8	0	73.2 ± 2.3	3.1 ± 0.5
	C8	17	48.2 ± 1.2	0.73 ± 0.01
**Urine samples**				
TB negative	A1	n/a	54.8 ± 5.5	ND
	A2	n/a	52 ± 0.8	ND
	A3	n/a	43 ± 0.2	ND
	A4	n/a	47.7 ± 5.1	ND
	A5	n/a	52.8 ± 5.7	ND
	A6	n/a	67.9 ± 9	ND
TB positive	A7	0	170.6 ± 4.7	8.8 ± 0.9
	A8	0	141.1 ± 2.6	6.3 ± 0.5
	A9	1	110 ± 1.1	3.6 ± 0.2
	A10	14	53.1 ± 3.1	ND

CFP10 concentration derived from concentration curves established for the BETA assay for serum and urine sample matrices independently (Fig. 3).

^a^Not detected.

**Figure 5:**
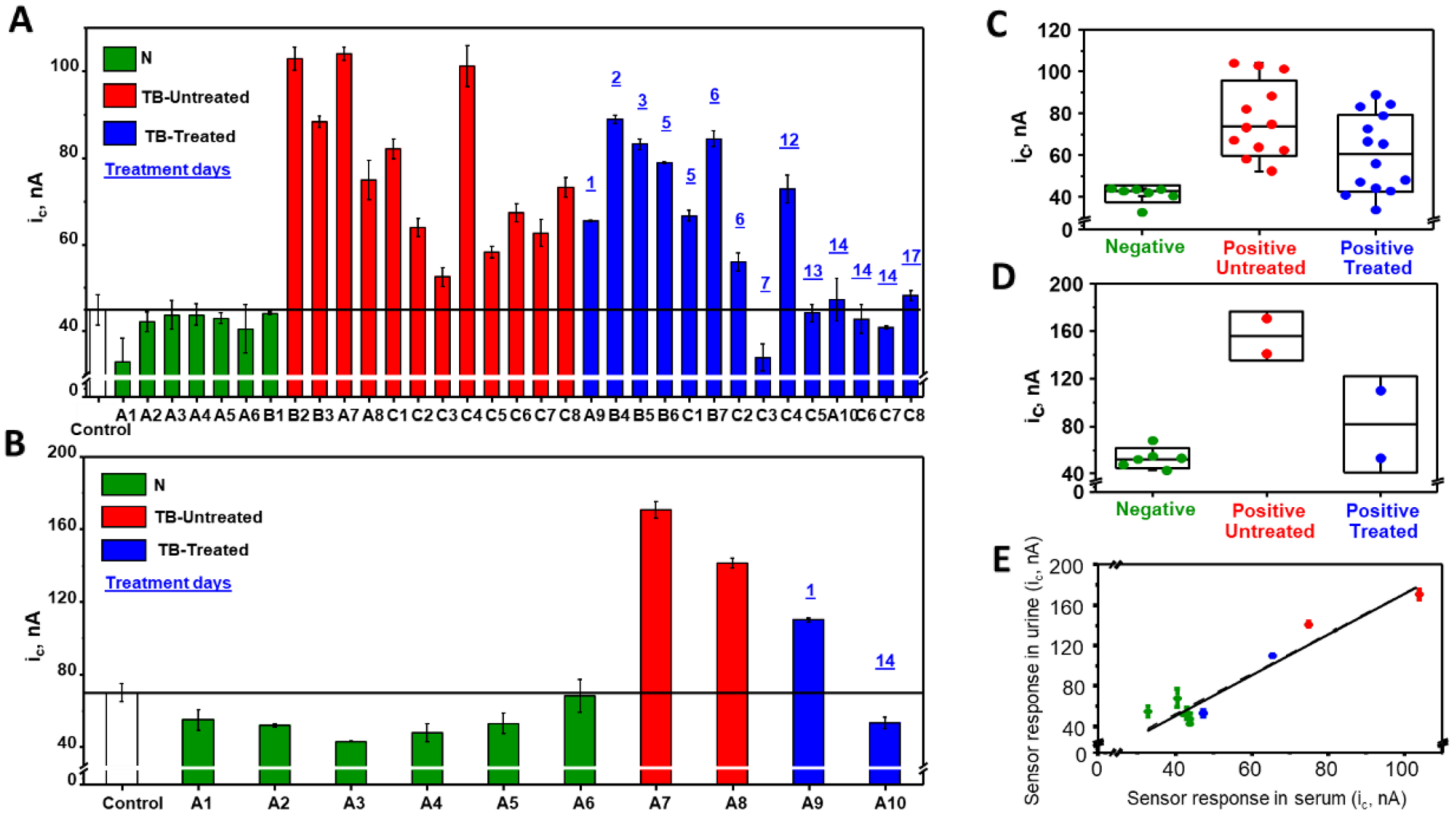
Amperometric response of the bioelectronic TB antigen (BETA) assay using clinical serum and urine samples from patients at risk for TB. Amperometric responses obtained from the BETA assay using clinical serum (**A**) and urine (**B**) samples. Results from ×3 replicates of negative control serum and urine (white bars), TB negative patient serum and urine (green bars), TB positive patient serum and urine with zero days of treatment before sample collection (TB—untreated: red bars) and TB positive patient serum and urine with 1–17 days of TB treatment before sample collection (TB—treated: blue bars). Black line represents tentative lower threshold for diagnosis of TB positivity. Box and whiskers plot of mean and interquartile range (IQR) of amperometric signals from clinical serum (**C**) and urine (**D**) from TB negative (green dots), untreated TB positive patients (red dots) and treated TB positive patients (blue dots). Correlation of amperometric responses obtained from paired serum and urine samples (**E**). Dots and error bars represent mean and standard deviation of three replicates. Cathodic current intensity (i_c_) measured in nA.

Amperometric responses for all clinical serum and urine samples evaluated (in triplicate) are presented in Fig. 5A,B. Based on the assumption that patients that had been treated for TB for more than a few days could have reduced CFP10 signal, we stratified microbiologically confirmed TB positive patients into those that had received zero days of treatment at time of sample collection (untreated), from those that had received between 1 and 17 days of treatment (treated). Mean signals from clinical serum samples from TB negative, TB positive (untreated), and TB positive (treated) individuals, were 41.4 nA, 77.6 nA and 60.9 nA respectively, see Fig. 5C. Mean amperometric signals from clinical urine samples from TB negative, TB positive (untreated) and TB positive (treated) patients, were 53.0 nA, 155.8 nA and 81.6 nA, respectively, see Fig. 5D. Amperometric responses from matched serum and urine samples from the same patient, regardless of treatment days, were highly correlated *Pearson’s r* = 0.95, p < 0.01 (Fig. 5E). Using concentration curves derived independently for serum and urine (Fig. 3), we estimated CFP10 concentrations in the clinical serum samples ranged from 1.1 to 5.9 nM in TB positive patients with < 7 days of treatment, while patients with ≥ 7 days of treatment had mostly undetectable levels of CFP10 (Table 2). CFP10 concentrations in urine appeared to be 1.5–2 times higher than in matched serum samples from the same patient.

### Treatment response

**Figure 6:**
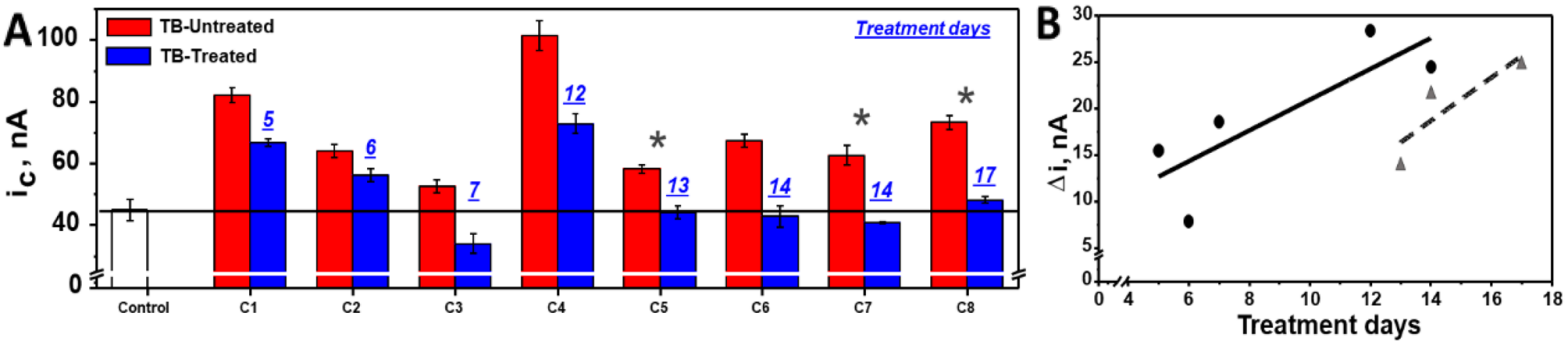
Amperometric results from a bioelectronic TB antigen (BETA) assay using paired clinical serum samples from the same patient over time to indicate effect of treatment days on signal. (**A**) Amperometric responses obtained from the BETA assay using serial clinical serum samples collected from the same TB positive patients on day zero before drug resistant TB treatment (TB-untreated: red bars) and after pre-set number of treatment days (TB—treated: blue bars). Results from ×3 replicates of negative control serum (white bar) and each clinical sample. Dashed black line represents tentative lower threshold for diagnosis of TB positivity. Blue numbers represent number of days of treatment. (**B**) Amperometric signal strength from paired, serial serum samples from individual patients, plotted against days of treatment and stratified by culture status of the patient at follow up time point. Round markers and solid line represent patients with AFB/culture negative sputa at follow up time point and triangle symbols and dashed line represent patients with AFB negative/culture positive sputa at follow up. Cathodic current intensity (i_c_) was measured in nA.

We evaluated pairs of serial samples from eight patients from study Cohort C (patient IDs labeled with “C” in Table 2) to examine treatment response in drug resistant TB patients. All eight patients were sputum smear and culture positive prior to treatment initiation. Follow-up samples were collected between 1 and 3 weeks after treatment initiation. Follow up samples from five of the patients were smear and culture negative at follow up while three patients were smear negative, but still culture positive at follow up. The BETA assay signals from patients at baseline (untreated) and follow-up (treated) can be seen in Fig. 6A. The difference between the amperometric signal at follow up and before treatment initiation (in triplicate) were plotted against number of treatment days (Fig. 6B), with separate slopes for patients that became culture negative (n = 5) and those that did not culture convert (n = 3). Controlling for follow-up culture status, days on treatment was significantly associated with decrease in CFP10 signal by linear regression (p = 0.023, R^2^ = 0.68), suggesting CFP10 reduction could be correlated with treatment response.

## Discussion

To our knowledge, this is the first report of a bioelectronic immunoassay being used to detect clinically relevant concentrations of CFP10 directly from unprocessed small-volume clinical serum or urine samples, indicating its potential as a novel, non-sputum-based diagnostic assay for active TB disease. Using our novel BETA assay we detected CFP10 antigen in concentrations ranging from 1.1 to 5.9 nM in serum and 3.6–8.8 nM in urine in all culture positive TB patients with less than one week of treatment. While prototype bioelectronics immunoassays for detecting CFP10 have been previously described, they have been limited to detection of CFP10 in highly processed clinical samples, contrived laboratory samples, sputum samples, or culture filtrate samples^26,27^. The BETA LOD we observed for detecting CFP10 in serum (0.4 nM) was an improvement over a previously reported bioelectronic assay using a graphene nanocomposite surface and nanoparticles (LOD = 1.4 nM), but was less sensitive than the previously described approach using quantum dots and gold-tagged antibodies (LOD = 0.03 nM)^21,22^. Additionally, it is important to note that other technologies, such as mass spectrometry have successfully detected *Mtb* CFP10 antigen peptides in the serum of TB positive patients at low nM to high pico molar concentrations^18,19^. Complex sample preparation protocols and costly instrumentation however preclude the utility of these technologies as point-of-care diagnostic solutions.

We also observed that estimated CFP10 concentrations in urine appeared to be up to two times higher than in the matched serum samples, indicating the BETA test might ultimately have greater sensitivity for detection of TB in urine than serum. This is consistent with other TB antigen studies evaluating concentrations of the *Mtb* antigens LAM and ESAT6 in TB patients, which reported higher antigen concentrations in urine compared to serum^11^. Our use of CFP10, an antigen secreted during bacterial replication may be detectable at higher concentrations compared to LAM which is present in bacterial cell wall.

In addition, BETA assay results of paired serum samples collected from drug resistant TB patients before and after treatment initiation suggested a correlation between decrease in CFP10 antigen signal and days on treatment, after controlling for final culture status. Although only a limited number of patients were evaluated, our data suggests that the BETA assay could not only be used as a point-of-care diagnostic to rapidly detect active TB disease, but it could also potentially be used to evaluate treatment response, allowing clinicians timely treatment regimen modification, in contrast to the reference standard, which is based on slow growing cultures that can take several weeks^2^. Further study, however, is needed to determine if antigen levels track lower and stay lower in those patients that ultimately have positive treatment outcomes, as well as more data on antigen levels over the course of treatment, to determine if we can identify threshold antigen levels that are indicative of a sustained response to treatment versus failure. Additional large scale field studies using a greater diversity of prospectively collected clinical samples are needed to evaluate the potential of the BETA assay to serve as both a highly sensitive diagnostic tool and a quantitative treatment response monitoring assay.

## Limitations

This study included only a limited number of clinical serum and urine samples as a proof-of-concept for the performance of the assay. While we endeavored to include samples from three independent study cohorts with age and sex diversity, and including both patients at risk for active TB and drug resistant disease, a more diverse and substantially larger set of clinical samples is needed to reliably estimate the sensitivity and specificity of the BETA assay. It is also important to recognize that although the assay was designed to detect circulating CFP10 produced during bacterial replication, regardless of infection site, only serum and urine samples from patients with pulmonary disease were evaluated in this study. Additional evaluation of samples from patients with extrapulmonary disease is necessary to determine its utility as a diagnostic for diverse presentations of disease. Likewise, our evaluation of a small set of serial samples from patients under treatment is only an indication of the potential of the BETA assay as a treatment monitoring tool and future studies are needed to determine CFP10 concentrations during treatment and their correlation with treatment response in order to set clinically relevant thresholds for interpretation.

## Summary/conclusions

Results from this proof-of-concept study indicate our novel BETA assay has potential to be used as a rapid, point-of-care diagnostic for active TB, as well as a near real-time TB treatment monitoring tool. The BETA assay is rapid (sample-to-answer in 90 min), requires only non-expert handling (droplet pipetting, washing, and drying), uses ultra-small unprocessed sample volumes (10 µL), has an estimated cost-of-goods for disposable consumables of below 5 USD, and can be read by a low-cost miniature potentiostat powered by a mobile phone application. Preliminary analytical performance metrics indicate that the current assay is highly sensitive and specific for identifying microbiologically confirmed TB positive patients from among individuals at risk of TB however further studies are needed to evaluate assay performance and to determine the potential utility of the assay as a point-of-care diagnostic for a wide range of TB disease states.

## Materials and methods

### Clinical sample acquisition

Clinical samples used in this study were obtained from three independent TB and drug resistant TB study cohorts from previously completed and ongoing pulmonary TB studies. Samples were collected under each study’s respective University of California San Diego IRB approved protocol, and bio-banked for future use. Informed consent was obtained at the time of specimen collection from all study participants. All study procedures were performed in accordance with approved protocols and relevant guidelines. In brief, participants in Cohort A were enrolled in an antigen diagnostic study (NIH- R01-AI141500) in San Diego. Serum and urine samples were collected from patients presenting for acute care who were assessed for TB disease and underwent standard reference testing with culture and/or GeneXpert. Due to enrollment delays, some TB positive participants in this cohort initiated treatment prior to sample collection. Samples from Cohort B were collected from patients recruited in Moldova as part of a study designed to identify blood-based biomarkers of progression from not infected to active TB infection (DoD-W81XWH-17-PRMRP-TTDA). Serum samples were collected from culture positive, index TB cases at treatment initiation, with some samples being collected post-treatment initiation. Samples included in Cohort C were collected from Moldovan participants enrolled in a study designed to evaluate a novel, molecular diagnostic for XDR-TB diagnosis (NIH-R01AI111435). Study participants consisted of individuals who were considered at risk for drug resistance. Initial serum samples were collected at enrollment, before treatment, and then again at set intervals during treatment. Bio-banked serum samples from all cohorts and urine from cohort A were stored at − 20 °C for weeks to years (3 years maximum) prior to subjecting them to BETA assay evaluation.

### BETA sensor fabrication and modification

Sensor fabrication and preparation methods are described in detail in the “Supplementary information”. BETA assay sensors were manufactured in batches using a previously developed proprietary photolithography-free masking method, with each sensor consisting of two rectangular 21 mm^2^ area electrodes on a plastic substrate to create the working electrode (WE) and a joint reference/counter electrode (RE/CE)^28^. WEs were modified by immobilizing commercial-grade, Mtb-specific, anti-CFP10 capture antibodies (CAb) onto the WE surface and “blocking” the surface to prevent non-specific binding (see additional fabrication and electrode modification details in the “Supplementary information” and Fig. S5).

### Sensor and protocol optimization

The BETA sensor and assay protocol were designed to achieve high analytical performance (sensitivity, specificity, and rapid time-to-result) with low-cost consumables. Particular attention was given to modification of the sputtered gold surface to ensure negligible non-specific adsorption effects, essential for high selectivity and short incubation time. Optimization of sensor and assay parameters were serially evaluated using chronoamperometry and Electrochemical Impedance Spectroscopy (EIS) and is described in more detail the “Supplementary information”.

### Assay calibration

As the assay performance of bioelectronic immunoassays can be highly dependent on the biochemical makeup of the sample matrix, we established calibration curves first in phosphate buffer (PB) as sample matrix, then in serum and urine independently. For calibration of the assay in PB, we used concentration standards of recombinant *Mtb* CFP10 antigen (ImmunoDx, MA) spiked into PB (0, 10, 50, 100 and 250 nM). For calibration of the assay for use with serum and urine sample matrices, *Mtb* CFP10 antigen was spiked in increasing concentrations (0, 1, 5, 10, 25 nM) into commercially acquired negative control, charcoal stripped (4X) human serum (Biochemed, VA) and pooled human urine (MyBioSource, CA).

### Sensor reproducibility and stability

Reproducibility of the optimized BETA assay was evaluated by comparing chronoamperometric measurements from 12 optimized BETA sensors, batch-fabricated and run using buffer (n = 6 replicates), and buffer spiked with 100 nM CFP10 antigen (n = 6). Storage stability of the sensors with bound CAb was assessed by batch-fabricating sensors and binding the CAb, then storing them at 4ºC and testing sensors on seven separate days over 30 days. Assays were run in triplicate with 100 nM *Mtb* CFP10 spiked into PB and amperometric readouts were averaged and recorded.

### Final assay protocol

The final prototype assay, optimized for serum and represented in a simplified schematic (Fig. 1A,B) is as follows: (1) 10 µL of serum was deposited on the modified WE and incubated for 45 min to bind CFP10 antigen to the CAb; (2) a PB washing step (repeated three times) was used to remove sample matrix and stop the immunoreaction; (3) 10 µL of commercial-grade, horseradish peroxidase (HRP) labeled anti-CFP10 detector antibody (HRP-DAb) solution was then placed on the WE and incubated for 45 min to sandwich the CFP10 between the CAb and DAb on the WE; (4) a 0.05% SDS washing step (repeated three times), and another PB washing step (repeated three times) was used to remove unbound HRP-DAb; (5) the BETA sensor was then inserted into an “EmStat3 Blue” potentiometer, attached to a laptop running “PSTrace” software (Palmsens, The Netherlands); (6) a 20 µL drop of 3,3′,5,5′-tetramethylbenzidine (TMB)/H_2_O_2_ was placed over both electrodes, and a cathodic current of − 0.1 V was applied and the amperometric signal was recorded for 2.5 min^29^. The current measured from the potentiometer corresponded to the reduction of the oxidized TMB, generated by the coupled reduction of H_2_O_2_ catalyzed by the HRP tag on the DAb, which was directly proportional to the amount of the HRP-tagged DAb bound to the sensor, corresponding to the concentration of CFP10 in the sample.

### Statistical analysis

Clinical samples were evaluated in triplicate; error bars were estimated as standard deviation of the three replicates. Limit of Detection (LOD) was estimated using the 3S_b_/m criterion, where S_b_ is the standard deviation for ten blank signal measurements and m is the slope value of the calibration plot. EIS data from sensor and protocol optimization was processed into Nyquist curves using “Gamry Echem Analyst” (Gamry, PA). Linear regression analyses were performed and concentration curves generated using “STATA 15” (College Station, TX) and “Origin” (OriginLab, MA). Pearson’s *r* was used to assess correlation between paired urine and serum amperometric responses.

## Supplementary Information

Supplementary Information.

## Data Availability

Individual level CFP10 concentration results are included in Table 2 and metrics for the assay development are included in the supplemental file.
