# Supplementary material for: Detection and quantification of Mycobacterium tuberculosis antigen CFP10 in serum and urine for the rapid diagnosis of active tuberculosis disease
Source: Sci Rep. 2021 Sep 28;11:19193. doi: 10.1038/s41598-021-98471-1 (PMC8478938; doi:10.1038/s41598-021-98471-1)
Supplement: Supplementary file 1 — Supplementary Information. [file 41598_2021_98471_MOESM1_ESM.pdf]

Supplementary Information

Title: Detection and quantification of *Mycobacterium tuberculosis* antigen CFP10 in serum and urine for the rapid diagnosis of active tuberculosis disease

Authors: Marva Seifert[1,3], Eva Vargas[2,3], Victor Ruiz-Valdepeñas Montiel[2], Joseph Wang[2,4], Timothy C Rodwell[1,4], Antonino Catanzaro[1,4]

Affiliation, address and Degree
[1] Division of Pulmonary, Critical Care and Sleep Medicine, Department of Medicine, University of California San Diego, 9500 Gilman Drive, La Jolla, CA 92093 (M Seifert PhD, TC Rodwell MD, A Catanzaro MD)
[2] Department of Nanoengineering, University California San Diego, 9500 Gilman Drive, La Jolla, CA 92093 (E Vargas PhD, V Ruiz-Valdepeñas Montiel PhD, J Wang PhD)

Author Contribution:
[3] Co-first authors, contributed equally
[4] Co-senior authors, contributed equally

Bioelectronic TB (BET) Assay Sensor Fabrication and Modification Details

BET assay sensors were manufactured in batches using a proprietary photolithography-free masking method. First, a Cricut machine was used generate the rectangular electrode patterns on the laminated protective cover of a polyethylene terephthalate glycol (PETG) plastic sheet (Small Parts Inc.). Then a "Denton Discovery 18 Sputter System" (Denton Vacuum, NJ), under direct current and with Argon gas, was used to sputter deposit Cr and then Au onto the PETG substrate. Each completed sensor consisted of two rectangular $21mm^2$ area electrodes: a working electrode (WE) and a joint reference/counter electrode (RE/CE).[1] The electrodes were immersed overnight at 4° C in a 0.1 mM MUA/1.0 mM MCH solution to form a stable thiol self-assembly monolayer (SAM). The electrodes were then washed with pure ethanol and water. The resulting SAM-functionalized WEs were incubated with 10 µL of a 0.4 M EDC/0.1 M NHS solution to activate the terminal carboxylic groups. After washing three times with phosphate buffer (PB), the electrodes were dried with a compressed air gun. After sensor fabrication, the WEs were modified by covalently attaching anti-CFP10 antibodies or capture antibodies (CAb) on the electrode surface by incubating the electrodes with 10µL of 100µg/mL CFP10 CAb solution for 45 minutes. The electrodes were again washed three times with PB and dried with a compressed air gun. Remaining unreacted carboxylic groups were deactivated by incubating the WE with 10 µL of ethanolamine solution for 30 min. After a final PB wash and dry, the CAb-modified WE sensors were stored at 4 °C until use.

Sensor Optimization and Testing

High sensitivity detection capability was achieved by comprehensive study and optimization of all the experimental variables affecting the BET antibody-modified sensor preparation and the bioassay protocol. These included composition of the binary self-assembled monolayer (SAM); concentrations of the 11-mercaptoundecanoic acid (MUA), 6-mercapto-1-hexanol (MCH) and ethanolamine reagents used to prevent non-specific adsorption; CAb and HRP-tagged Mtb CFP10 detector antibody (HRP-DAb) concentration; number of assay steps; solution composition for enzymatic labeling and incubation; and incubation times for CAb, CFP10 antigen and HRP-DAb binding. In order to identify the optimal approach, all of the sequential surface modification steps performed, from initial functionalization of the gold electrode to the final HRP-tag capturing on the sensor were monitored by Electrochemical Impedance Spectroscopy (EIS) using a Gamry "Interface 1010E" instrument (Gamry, PA) (See Figure S2).

Figure S1. Results of optimization experiments for critical bioelectronic TB (BET) sensor and assay parameters in phosphate buffer. Chronoamperometric signals obtained from evaluated parameters of a novel BET assay using phosphate buffer as a negative control or Blank (labeled "B" and shown with white bars) and phosphate buffer spiked with 100 nM of Mtb CFP10 as a positive control or Signal (labeled "S" and shown with grey bars). Graphs show change in signal-to-blank (S/B) ratio (red lines) with different parameters: a) MUA concentration; b) MCH concentration; c) Cab concentration; d) Ethanolamine concentration; e) HRP-DAb concentration; f) Number of incubation steps; g) HRP-DAb incubation media; h) CAb incubation time; i) antigen target incubation time; j) HRP-DAb incubation time. Error bars were estimated as the standard deviation of three replicates.

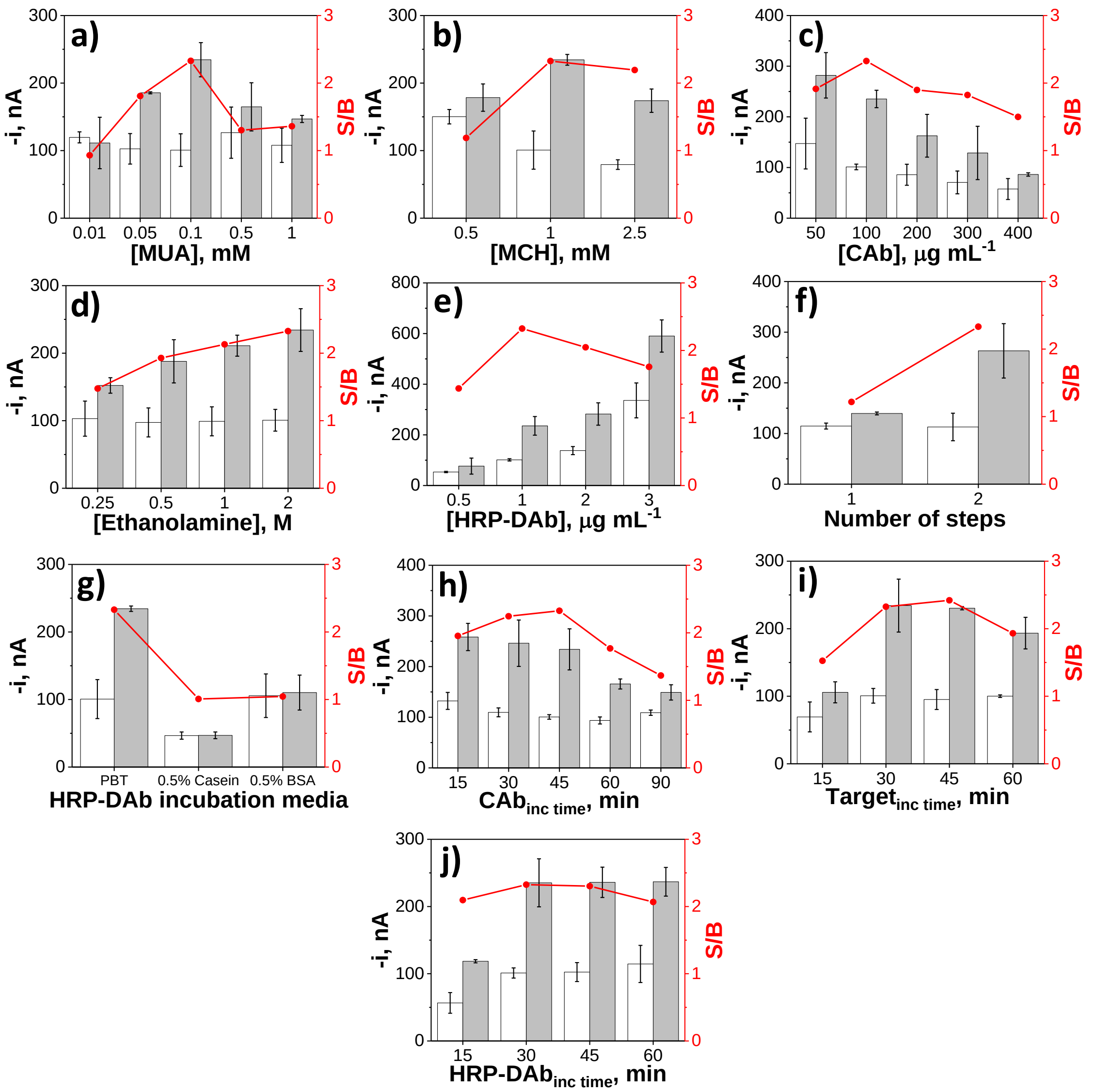

Figure S2. Electrochemical performance and characterization of the Bioelectronic TB (BET) sensor based on CFP10 spiked into negative control phosphate buffer. Nyquist curves resulting from the electrochemical characterization of the sequential modification steps used to develop the BET immunoassay.

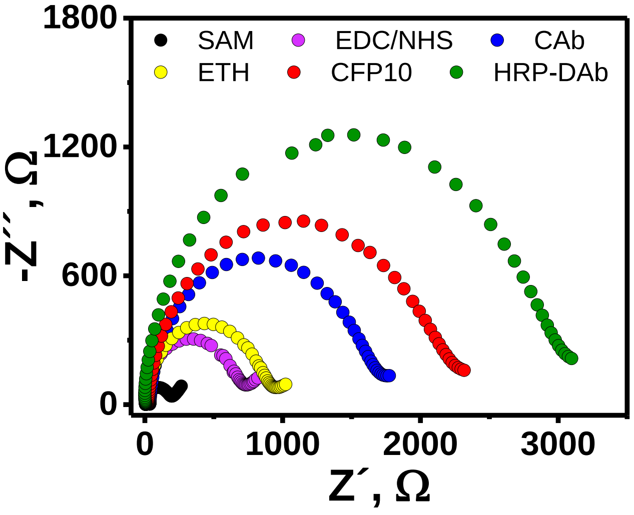

Figure S3. Results of optimization experiments for critical bioelectronic TB (BET) assay parameters in serum. Chronoamperometric signals obtained from evaluated parameters of a novel BET assay using charcoal stripped (4X) human serum as a negative control or Blank (labeled "B" and shown with white bars) and charcoal stripped serum spiked with 25 nM of Mtb CFP10 as a positive control or Signal (labeled "S" and shown with grey bars). Graphs show change in signal-to-blank (S/B) ratio (red lines) with different assay parameters: a) CAb concentration; b) Number of incubation steps; c) antigen target incubation time; d) HRP-DAb concentration; e) HRP-DAb incubation time. Error bars were estimated as the standard deviation of three replicates.

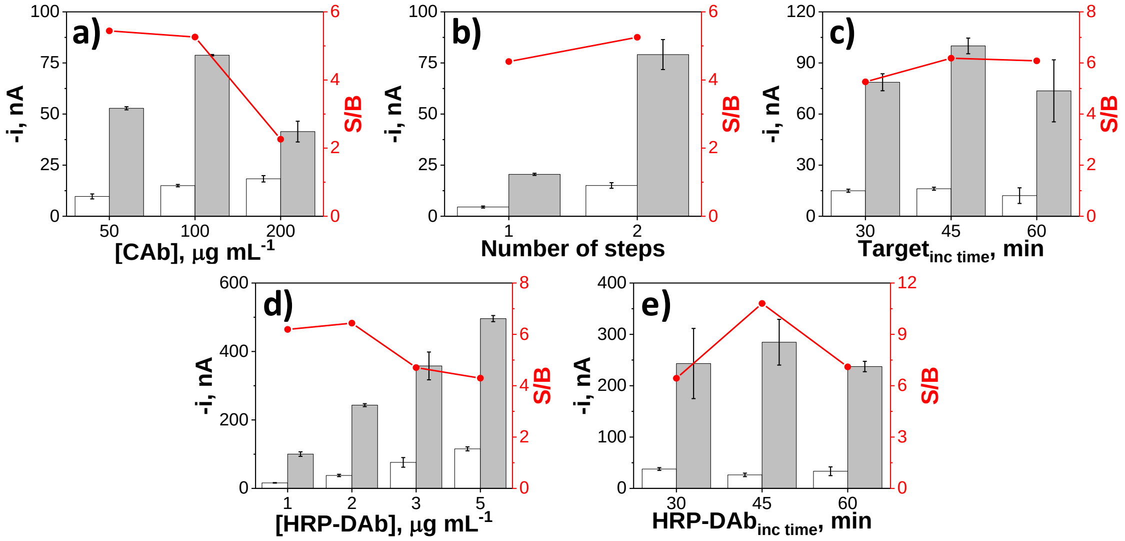

Table S1. Evaluated ranges and final optimal sensor modifications and assay run parameters selected for the bioelectronic TB (BET) assay in buffer and serum matrices.

Sensor Modification/ Assay Run Parameter	Optimized in buffer		Re-optimized in serum	
	Evaluated range	Selected value	Evaluated range	Selected value
**MUA concentration (mM)**	0.01–1	0.1	–	
**MCH concentration (mM)**	0.1–2.5	1.0	–	
**Cab concentration (µg mL^{-1})**	50–400	100	50–200	100
**Cab incubation time (min)**	15–90	45	–	
**Number of incubation steps**	1–2	2	1–2	2
**Target incubation time (min)**	15–60	30	30 60	45
**Ethanolamine concentration (M)**	0.25–2	2	–	
**Ethanolamine incubation time (min)**	–	30	–	
**HRP-DAb concentration (µg mL^{-1})**	0.5–3	1	1–5	2
**HRP-DAb incubation time (min)**	15–60	30	30–60	45
**HRP-DAb incubation media**	PBT<br>0.5% Casein<br>0.5% BSA	PBT	–	

MUA=mercaptoundecanoic acid, MCH=6-mercapto-1-hexanol, CAb=capture antibody, HRP-DAb=HRP tagged detector antibody, PBT=phosphate buffer + tween BSA= bovine serum albumin

Figure S4. Background signals observed with the Bioelectronic TB (BET) assay from negative control serum and urine matrices, and effect of blocking. a) Background chronoamperometric responses of the BET assay in the absence of CFP10, obtained from negative controls consisting of buffer, commercially acquired charcoal stripped (4X) human serum and urine matrices. b) Chronoamperometric responses obtained from the buffer-optimized BET immunoassay protocol compared to the responses after applying a blocking step to the sensor fabrication; using undiluted (i, ii) or diluted (iii) commercial horse serum for 30 (i, iii) or 15 minutes (ii) incubation on the sensor. Measurements were made from undiluted negative control urine samples (B – orange/white stripped bars) and urine spiked with 10 nM of Mtb CFP10 antigen standard (S - orange bars). The blue lines and diamonds indicate S/B ratio for each positive/negative control pair. Error bars were estimated as the standard deviation of three replicates for each.

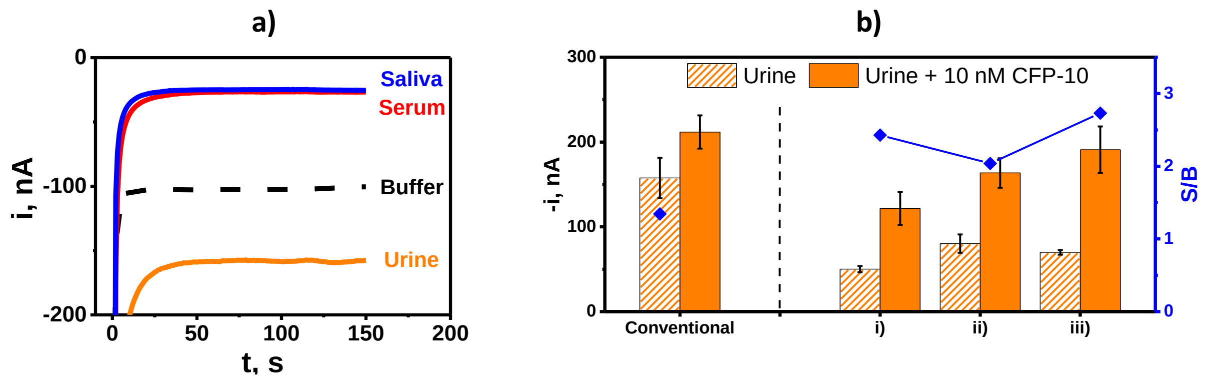

Figure S5. Bioelectronic TB (BET) Assay sensor fabrication protocol. Sequential modification steps of the electrode surface for the SAM formation, carboxylic groups activation, immobilization of the CAb, and blocking reaction with ethanolamine.

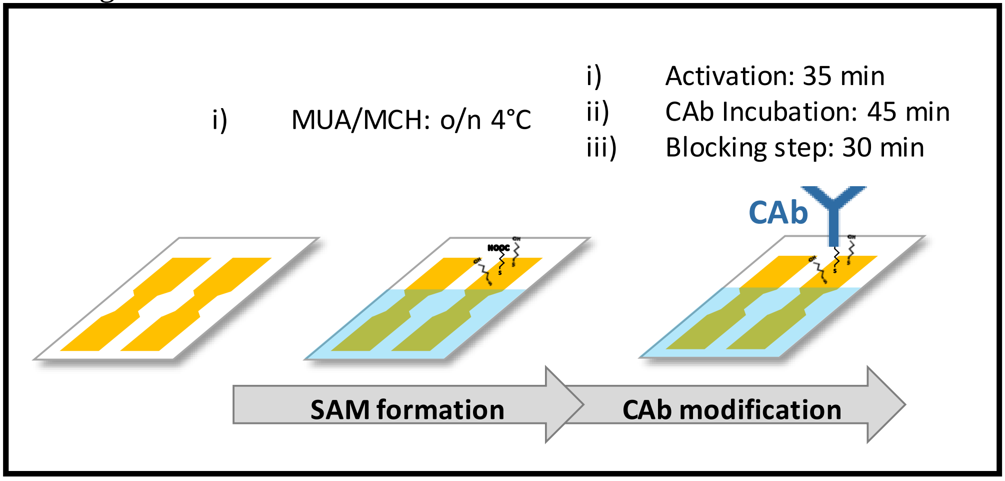

References:

1. Vargas, E. *et al.* Enzymatic/Immunoassay Dual-Biomarker Sensing Chip: Towards Decentralized Insulin/Glucose Detection. *Angew Chem Int Ed Engl*, doi:10.1002/anie.201902664 (2019).
